# GIPC2 interacts with Fzd7 to promote prostate cancer metastasis by activating WNT signaling

**DOI:** 10.1038/s41388-022-02255-4

**Published:** 2022-03-28

**Authors:** Liang Wang, Jiayi Wang, Xiaolin Yin, Xin Guan, Ying Li, Chenqi Xin, Jing Liu

**Affiliations:** 1 Stem Cell Clinical Research Center, National Joint Engineering Laboratory, Regenerative Medicine Center, The First Affiliated Hospital of Dalian Medical University, No. 193, Lianhe Road, Shahekou District, Dalian City, Liaoning Province 116011 PR China; 2 Dalian Innovation Institute of Stem Cell and Precision Medicine, No. 57, Xinda Street, Dalian High-Tech Park, Dalian City, Liaoning Province 116023 PR China

**Keywords:** Oncogenes, Epigenetics, Diagnostic markers

## Abstract

Prostate cancer (PCa) causes significant mortality and morbidity, with advanced metastasis. WNT signaling is a promising therapeutic target for metastatic PCa. GIPC2 is a GIPC1 paralog involved in WNT signaling pathways associated with tumor progression, but its role in PCa metastasis remains unclear. Herein, we demonstrated that high GIPC2 expression in PCa tissues was significantly associated with distant metastasis and poor prognosis. Functional studies demonstrated that high GIPC2 expression due to CpG-island demethylation promoted increased metastatic capabilities of PCa cells. Conversely, silencing GIPC2 expression significantly inhibited PCa metastasis in vitro and in vivo. Furthermore, GIPC2 directly bound the WNT co-receptor Fzd7 through its PDZ domain, which enabled activation of WNT-β-catenin cascades, thereby stimulating PCa metastasis. Interestingly, GIPC2 protein was also identified as a component of exosomes and that it robustly stimulated PCa adhesion, invasion, and migration. The presence of GIPC2 in tumor-derived exosomes and ability to impact the behavior of tumor cells suggest that GIPC2 is a novel epigenetic oncogene involved in PCa metastasis. Our findings identified GIPC2 as a novel exosomal molecule associated with WNT signaling and may represent a potential therapeutic target and biomarker for metastatic PCa.

## Introduction

Prostate cancer (PCa) is a prevalent cancer affecting men worldwide characterized by significant mortality and morbidity, advanced metastasis, and age-related onset [1–4]. Although clinical therapies for metastasis are continually being developed, nearly two-thirds of patients who die from PCa, experience metabolic progression [1, 5, 6]. The molecular mechanisms underlying PCa metastasis remain unclear. PCa metastasis is a multi-step, complex process involving specific changes in master regulator genes that activate the metastatic cascades [7–9]. Genetic and epigenetic analyses have suggested that epigenetic alterations in oncogenes, tumor suppressors, and metabolism-related genes closely associating with the WNT-signaling pathway may drive metastatic PCa (mPCa) [10–13]. Therefore, molecules involved in the WNT-signaling pathway should be thoroughly investigated [14].

WNT signaling is an important contributor to PCa-related processes, such as cell proliferation, migration, differentiation, and self-renewal [14, 15]. Protein–protein interactions in the WNT-signaling pathway lead to subsequent activation of downstream cascades in PCa [14–16]. WNT binds co-receptors such as the transmembrane frizzled (FZD), low-density lipoprotein, and tyrosine-protein kinase transmembrane (ROR) receptors, as well as tyrosine-protein kinase RYK, to activate canonical (β-catenin-dependent) and noncanonical (β-catenin-independent) signals, which are generally biphasic and modulated by functional interactions with tumor-suppressor or tumor-promotor proteins [14, 17]. Furthermore, preclinical data have identified potential inhibitors able to bind to WNT receptor complexes to prevent PCa metastasis [15, 18].

GAIP-interacting protein, potentially participates in the WNT-signaling pathway. The C-terminus (GIPC) family contains three members (GIPC1, GIPC2, and GIPC3) that contain GIPC homology 1 (GH1), PDZ, and GH2 domains with functional similarity and evolutionary conservation, and GIPCs participate in processes driving familial hearing loss and cancer, such as planar cell polarity, cytokinesis, cell proliferation, and migration [19, 20]. The roles of GIPC1 in certain cancers have been comprehensively characterized, especially in terms of transmembrane trafficking and cellular migration [19, 21]. GIPC1 can be upregulated as a conditional oncogene that promotes cell proliferation and survival in breast cancer, ovarian cancer, and pancreatic cancer [22, 23], but it is downregulated in cervical cancer. GIPC3 germline mutations have been associated with hereditary deafness. However, the functions of GIPC2 remain poorly understood.

GIPC2 encodes a 315-amino acid protein that is 62% identical to GIPC1 and is considered a paralog of GIPC1 that promotes proliferation and invasion in tumor metastasis [19]. Theoretically, GIPC2 may perform similar functions as GIPC1 in tumorigenesis [19, 21]. Indeed, GIPC2 is upregulated in gastric cancer, but downregulated in kidney cancer, adrenocortical carcinoma, and acute lymphocytic leukemia (ALL) [19, 24, 25]. Furthermore, GIPC2 promoter hypermethylation has been detected in patients and cell lines, consistent with its suggested potential role as an epigenetic tumor suppressor in ALL [24]. A *Xenopus* ortholog of Kermit1 and GIPCs have been reported to interact with WNT receptors, including Frizzled-3 (Fzd3) and Fzd7 [15, 26]. Moreover, we previously reported that GIPC2 expression paralleled that of BMI-1, a key epigenetic regulator upstream of the WNT pathway in PCa [27]. Furthermore, GIPC2 is an endocrine-specific conditional tumor suppressor, whose inactivation is associated with promoter hypermethylation in sporadic and hereditary pheochromocytoma/paraganglioma (PPGL) via inactivation of p27 and suppression of PPGL cell proliferation and tumor growth both in vitro and in vivo [28]. Thus, these findings raise the question of what role methylated GIPC2 is playing in the WNT-signaling pathway in PCa.

Herein, we identified a novel putative oncogenic gene GIPC2 mainly expressed in exosome, which was regulated by epigenetics in PCa tumorigenesis. Functional studies unravel the role of GIPC2 in promoting PCa metastasis without affecting cell proliferation or apoptosis in vitro and in vivo. Mechanism investigation and mass spectrometry (MS) experiments revealed that GIPC2 interacted with WNT receptor Fzd7 through its PDZ domain and showed the oncogenic activity by activating WNT-β-catenin regulatory axis during PCa metastasis. Moreover, the exosomal GIPC2 positively correlated with PCa metastasis, suggesting its potential as an effective diagnostic biomarker of mPCa. Our study highlights the oncogenic function of GIPC2 in PCa metastasis, and indicates its promising clinical significance as a non-invasive biomarker for mPCa diagnosis.

## Results

### Increased GIPC2 expression in PCa cell lines and clinical patients

**Fig. 1:**
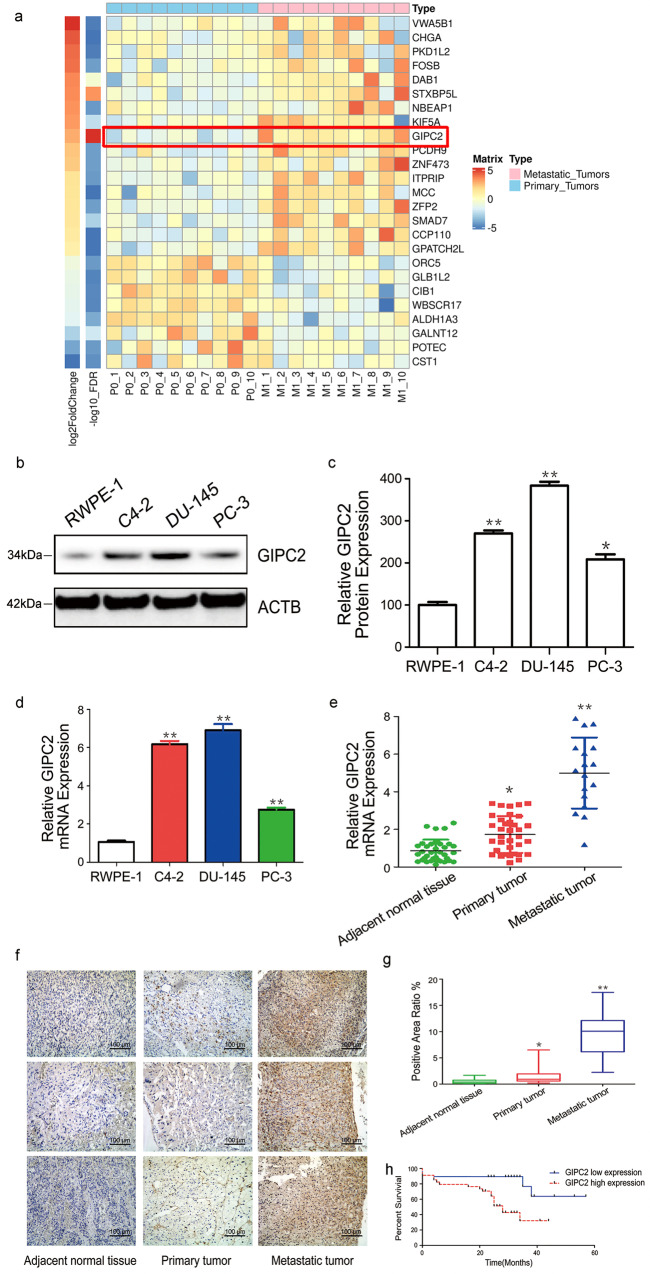
Significant GIPC2 upregulation in metastatic prostate cancer. **a** 60 ng of RNA from PCa patients was utilized to assess RNA sequencing data for gene expression (*n* = 20) comprised of patients with de novo metastatic (clinical stage M1, *n* = 10) as well as presumed localized (clinical stage P0, *n* = 10) tumors. Heatmap shows 25 significantly (FDR ≤ 0.05) differentially expressed genes (ranked by log2(fold change) value) across two comparisons (primary tumors vs. metastatic tumors). The log2(fold change) and -log10(FDR) were shown on the left side of the heatmap. **b**–**d** Four prostate cancer cell lines were incubated in 1640 medium with 10% FBS, and total protein (**b**, **c**) or RNA (**d**) was extracted. Western blot analysis was performed to analyze GIPC2 protein expression in each cell line, whereas real-time RT-PCR was performed to analyze GIPC2 mRNA expression. The protein- and mRNA-expression levels were normalized to those of ACTB. **e** GIPC2 mRNA expression in primary prostate cancer tissues (*n* = 36), metastatic prostate cancer tissues (*n* = 17), and normal adjacent tissues (*n* = 36), these tissues all derived from 53 patients. Relative to normal adjacent tissues, GIPC2 trended toward decreased expression in primary prostate cancer tissues and displayed significantly increased expression in metastatic prostate cancer tissues (***p < 0.0001, two-sided Mann–Whitney test). **f** Evaluation of GIPC2 staining in prostate cancer tissues. GIPC2 IHC staining was performed with primary prostate cancer and metastatic prostate cancer tissues, as well as normal adjacent tissues, and analyzed statistically. **g** Positive IHC-staining areas in each group were analyzed using Image J software. The data shown represent the mean ± SD of three independent experiments. Scale bar, 100 µm. **p < 0.01. **h** Overall survival analysis versus GIPC2 expression.

In order to investigate the target genes in mPCa progression, we first screened a series of 20 cases clinically diagnosed with primary (*n* = 10, P0) or metastatic tumors (*n* = 10, M1) representing the spectrum of PCa to identify promising candidate genes contributing to metastasis. We identified 25 genes that were differentially expressed including GIPC2 with the highest significant false discovery rate (FDR) *Q*-value (Fig. 1a). Upregulation of GIPC2 acts as a oncogene that promotes cell proliferation and survival, whereas downregulation of GIPC2 acts as a tumor suppressor. Thus, we assessed the expression of GIPC2 in the normal prostate cell line (RWPE-1), in mPCa cell lines (C4-2, DU145, and PC3), and in primary or mPCa tissues, in order to determine the effects of GIPC2 expression. Assessment of the relative expression levels of GIPC2 mRNA in prostate tissues showed GIPC2 expression to be more prominently increased in mPCa (mean relative expression 280.06 ± 240.1) than in primary tumors (mean relative expression 41.36 ± 16.81). Increased GIPC2 protein- and mRNA-expression levels were also confirmed in PCa cells (Fig. 1b–d). Moreover, RT-PCR results showed that GIPC2 expression was highest in mPCa samples (Fig. 1e). We further validated GIPC2 protein levels in normal tissues adjacent to primary and mPCa tumors by IHC, and clinical specimens from a PCa cohort (Supplementary Tables S1 and S2). Immunopositivity in clinical samples was assessed as the percentage of GIPC2-positive areas. Positive staining was predominantly observed in metastatic neoplasms from bones (200×) (Fig. 1e). Adjacent normal tissue and primary PCa tumors showed significantly less GIPC2 staining (Fig. 1f, g, Supplementary Fig. S1). Approximately 82.4% of mPCa contained medium-to-high cytoplasmic GIPC2 expression. We also analyzed correlations between GIPC2 expression and PCa clinical parameters (survival, Gleason scores, and diagnostic biochemistry markers such as Pre-op TPSA and Pre-op F/TPSA) (Fig. 1h and Supplementary Figure S2–S5). Re-analysis of the publicly available dataset from the GEO (GSE147250) and TCGA (phs000178), Database, showed GIPC2 was upregulated in metastatic than primary PCa (Supplementary Figs. S6, S7 and Supplementary Table S3). Collectively, the results suggested that GIPC2 expression was significantly higher in mPCa and that GIPC2 was upregulated in mPCa.

### GIPC2 promoter demethylation resulted in increased GIPC2 expression in PCa

**Fig. 2:**
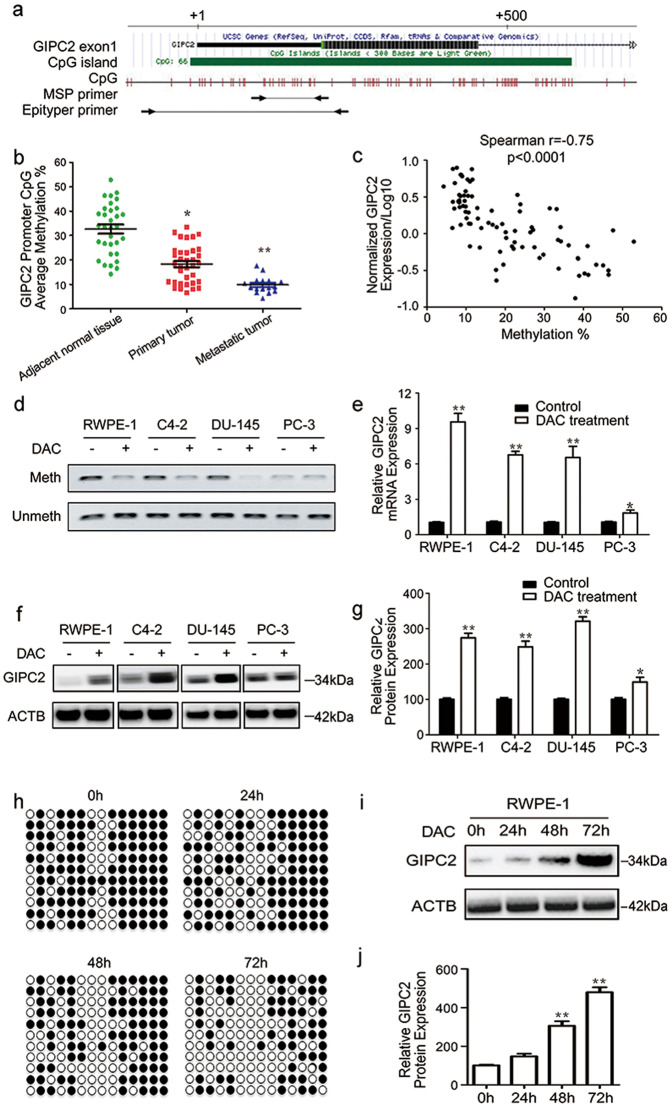
GIPC2 promotor methylation regulated GIPC2 expression in prostate cancer. **a** Schematic representation of the locations of CpG islands. The red bar shows the CpG sites. The corresponding numbers indicate the genomic locations designated in the UCSC database (hg38). **b** The promoter-methylation ratios of GIPC2 in prostate cancer samples were detected by EpiTYPER methylation analysis. The methylation ratios were significantly higher in normal adjacent tissues than in prostate cancer tumors. **c** Correlation between methylation of GIPC2-promoter CpG islands and their expression levels in all samples (*n* = 89). **d** The promoter-methylation ratios of GIPC2 were determined by MSP in four cell lines treated with or without DAC. Unmethylated (Unmeth) and methylated (Meth) PCR products were detected. After DAC treatment, GIPC2 mRNA- and protein-expression levels were detected using RT-PCR (**e**) and western blotting (**f**, **g**). The error bars represent the mean ± SD. **h** Effect of DAC expression on the methylation status of the GIPC2 promoter. DNA from control- or DAC-treated RWPE-1 cells was collected at the indicated time points, cloned, and sequenced to detect CpG-island methylation at the GIPC2 promoter. A summary of bisulfite-treated gDNA-sequencing results from RWPE-1 cells treated with DAC for increasing times is shown, where the amplified region contained 14 CpG sites (represented by circles located along the region) were analyzed by DNA sequencing. The black and white circles represent methylated and unmethylated CpG dinucleotides, respectively. Each line represents the DNA sequence of a random clone, for which the black and white circles represent unmethylated and methylated CpG sites in these regions, respectively. **i**, **j** RWPE-1 cells were treated with DAC, and GIPC2 protein expression was detected at 0, 24, 48, or 72 h.

CpG methylation modulates GIPC2 expression in cancer cells [24]. Therefore, we hypothesized that promotor demethylation could activate GIPC2 expression. To test this hypothesis, we used the EpiTYPER MassARRAY System (Sequenom) for quantitative DNA-methylation analysis. GIPC2 promoter-methylation levels indicated aberrant demethylation in mPCa versus adjacent normal tissues (Fig. 2a, b). GIPC2 promoter methylation negatively correlated (*p* < 0.01) with GIPC2 expression (Fig. 2c). We observed low GIPC2 expression in localized tumors or the normal prostate cell line, RWPE-1, and increased expression in metastatic tumor or cell line (Fig. 1b, c). To determine whether GIPC2 demethylation promoted decreased mRNA expression, we treated RWPE-1, C4-2, Du145, and PC3 cells with the DNMT1 inhibitor, DAC. Using methylation-specific PCR (MSP), DAC treatment decreased methylation levels, and three cell lines (RWPE-1, C4-2, and Du145) demonstrated gradual demethylation and significant increases in GIPC2 mRNA and protein expression (Fig. 2d–g). We also detected CpG-island methylation at the GIPC2 promoter by DNA sequencing. To determine the relationship between GIPC2 promoter demethylation and GIPC2 expression, RWPE-1 cells were treated with DAC, and GIPC2 protein expression was assessed at 0, 24, 48, or 72 h. The results confirmed that GIPC2 promoter demethylation increased GIPC2 expression in PCa (Fig. 2h–j).

### GIPC2 did not influence PCa proliferation or apoptosis in vitro

**Fig. 3:**
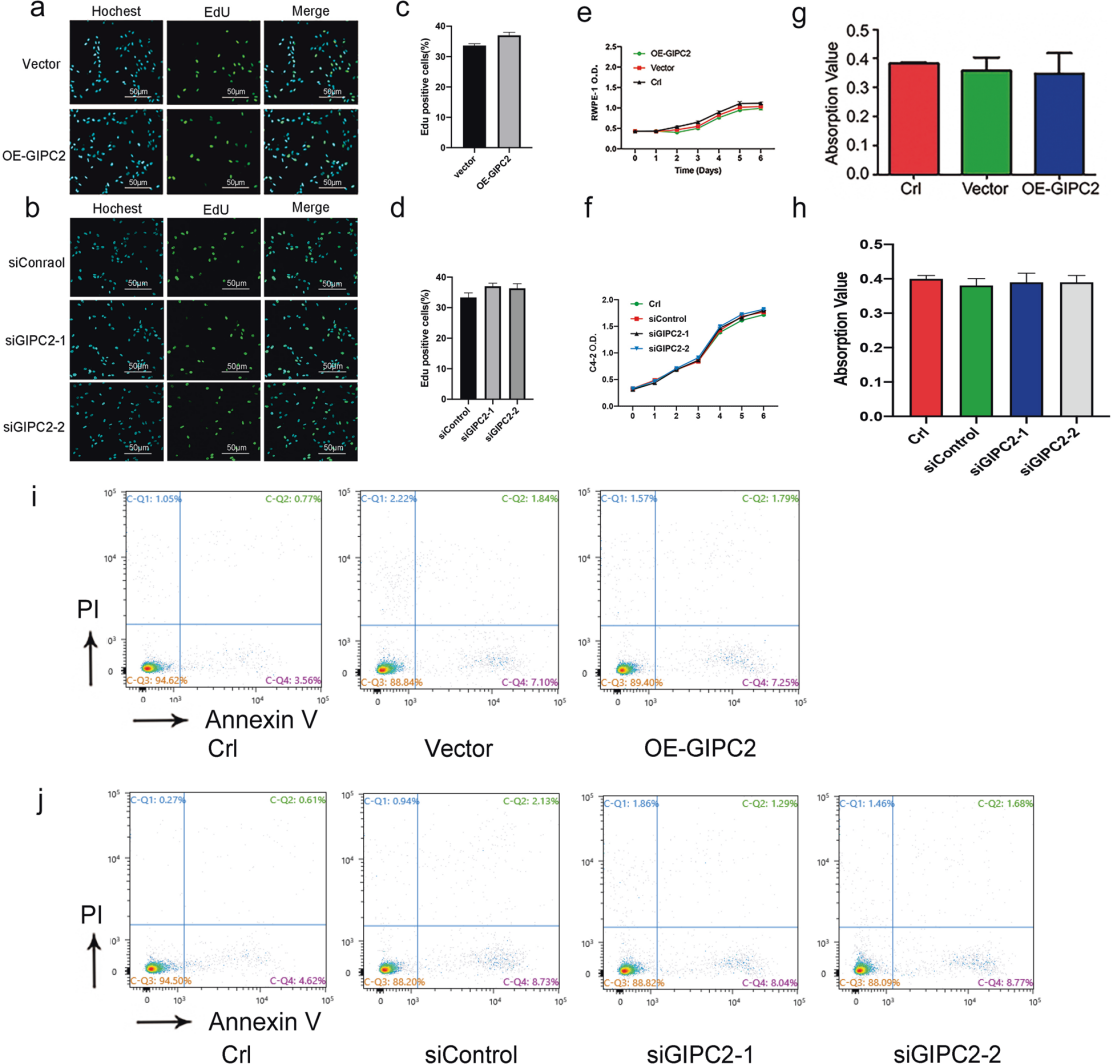
GIPC2 did not impact prostate cancer proliferation or apoptosis in vitro. **a**–**d** EdU staining and (**e**, **f**) CCK8 assays were performed to evaluate the effect of GIPC2 on cell proliferation. RWPE-1 and C4-2 cells were transfected with pcDNA3.1-GIPC2 or siRNA of GIPC2 (siGIPC2-1/siGIPC2-2), and cell proliferation was detected by EdU staining and a cell-viability kit at different time points. Apoptosis was determined using a Cell Death Detection Kit (**g**, **h**) and FCM (**i**, **j**). GIPC2 overexpression in RWPE-1 cells and GIPC2 knockdown in C4-2 cells. All experiments were performed at least thrice and the data shown represent the mean ± SD. *p < 0.05, **p < 0.01, two-sided paired *t* test.

Based on our findings above, we asked whether GIPC2 exhibits the biological characteristics of an oncogene in vitro. Thus, we evaluated the effects of GIPC2 on cell viability. C4-2 cells were transfected with GIPC2 siRNA, and RWPE-1 cells were transfected with a GIPC2-overexpression vector. Unexpectedly, cell-viability assays revealed that GIPC2 did not affect cell proliferation in PCa cells (Fig. 3a–f, Supplementary Fig. S8a, b). Next, we evaluated the effects of GIPC2 on apoptosis in PCa cells by quantifying DNA fragmentation. Treatment and control groups exhibited statistically equivalent levels of apoptosis (Fig. 3g, h). These results were confirmed by flow cytometry (Fig. 3i, j).

### GIPC2 promoted PCa metastasis in vitro and in vivo

**Fig. 4:**
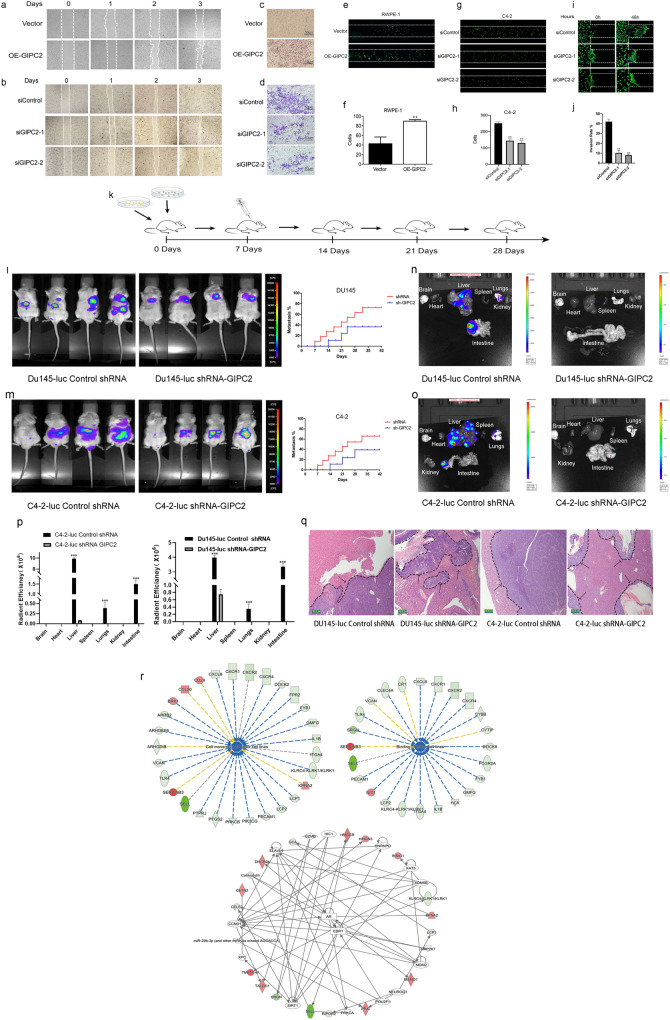
GIPC2 promoted prostate cancer motility and metastasis. **a**, **b** Cell-migration abilities were measured by performing wound-healing assays. Transfecting RWPE-1 and C4-2 cells with pcDNA-GIPC2 or siGIPC2-1/siGIPC2-2 attenuated the pro-migration and pro-invasion effects of GIPC2. After scratching a wound and removing the floating cells, prostate cancer cells (without treatment or control-treated) were tested in wound-scratch assays for 0–3 days. **c**–**j** Cell invasion was analyzed by performing transwell assays and the microfluidic platform. **c**, **d** Prostate cancer cells treated as described above were applied to transwell chambers coated with Matrigel and incubated for 24 h. **e**–**h** Representative fluorescent images of RWPE-1 and C4-2 cell adhesion (green: Calcein-AM). In these images, the endothelial barrier region was treated with 100 ng/ml EGF. Adhesion selectivity to EGF-stimulated was comparable for RWPE-1 and C4-2 cells, including GIPC2-overexpression RWPE-1 cells and GIPC2-knockdown C4-2 cells. **i**, **j** The microfluidic model consists of two independent microchannels, where C4-2 cells and 20% FBS were seeded. Between the two channels, 3D Matrigel was seeded to mimic the ECM. Invading cells were labeled with Calcein-AM (green). The data shown represent the mean ± SD. *p < 0.05; **p < 0.01. **k** Schematic representation of a mouse model of prostate cancer metastasis. **l**, **m** C4-2-luc Control or shRNA-GIPC2-1 and DU145-luc Control or shRNA-GIPC2-1 were suspended in 15 μL sterile PBS and used for intracardiac injections into male BALB/c nu/nu mice (*n* = 8 of each group, 32 mice in all). Bioluminescent images of metastatic tumors were monitored. Metastasis rates were determined according to the Kaplan–Meier method. *p < 0.05 between two groups. **n**–**p** The metastatic burden of different organs was quantified by monitored using an IVIS Lumina II (*n* = 3 of each group). Colored scale bars represent low (purple) to high (red) tumor burdens. **q** Paraffin sections of organs were stained with hematoxylin and eosin (H&E) and the tumor tissue is indicated by an arrow. **r** Upregulated and downregulated genes in GIPC2-overexpression RWPE-1 cells were imported into IPA. Through core analysis, the top associated networks and functions were found, and the first and the only meaningful pathway is shown. Red shading represents upregulated genes and green shading represents downregulated genes in the microarray.

Although cell proliferation and apoptosis are important biological processes in tumorigenesis, invasion and migration are believed to be more significant in mPCa pathogenesis [1]. Therefore, we hypothesized that GIPC2 in PCa could promote cell adhesion, invasion, and migration. A Transwell-based invasion and migration assay was established to quantitatively evaluate RWPE-1 invasion in vitro. Compared with controls, the average number of invading RWPE-1 cells increased after GIPC2 overexpression (Fig. 4a, c). Conversely, GIPC2 downregulation significantly decreased cell invasion (Fig. 4b, d). To confirm whether GIPC2 overexpression positively correlated with PC metastasis, we performed microfluidic assays, with PCa adhesion, invasion, and migration as the evaluation factors. Each specimen was divided into 30 layers for imaging and three equally spaced stacks were selected for quantitative cell counting. RPWE-1 cell adhesion increased significantly following GIPC2 overexpression (Fig. 4e, f). We also knocked down GIPC2 expression in C4-2 cells and, unsurprisingly, the adhesive capacities of C4-2 cells significantly decreased after GIPC2 downregulation (Fig. 4g, h). The migration distance was remarkably lower in GIPC2-downregulated cells compared to GIPC2 control cells (Fig. 4i, j).

To further characterize the effects of GIPC2 on metastasis in mice, we established C4-2 and DU145 cell lines with stably downregulated GIPC2, and implanted them into the left cardiac ventricle of male athymic nude mice (Fig. 4k). We monitored the bioluminescence emitted from cancer cells weekly to monitor metastatic growth of the prostate tumors. Compared with the control group, metastasis decreased in the GIPC2-knockdown group (Fig. 4l, m, Supplementary Figure S8c–e). Further, ex vivo imaging of organs was performed to determine the complete biodistribution pattern of cancer cells. This analysis showed weak cancer cell metastasis in the liver of the GIPC2-knockdown group (Fig. 4n–p, Supplementary Figure S8g, h). Pathological results showed that compared with GIPC2- knockdown, the control group presented marked metastasis in the liver (Fig. 4q, Supplementary Fig. S8f). Thus, decreased GIPC2 expression significantly inhibited PCa cell metastasis in mice.

To further understand and confirm GIPC2-mediated oncogenic functions in PCa metastatic progression, we used an mRNA-expression microarray (the Affymetrix Human HTA 2.0 array) to identify genes associated with GIPC2 expression. We next investigated what diseases and functions were impacted by GIPC2 overexpression in RWPE-1 cells. We input into ingenuity pathways analysis (IPA) all the 1264 genes that were significantly differentially expressed between GIPC2-overexpression and Control groups (Supplementary Table S4). Only 1128 of the 1264 genes differentially expressed (687 downregulated and 441 upregulated), mapped into the IPA knowledgebase. The top 270 significant genes were analyzed and enriched in cellular movement-related pathways (FC > |2| *p* < 0.01; Supplementary Table S4). This result suggested that GIPC2 overexpression exerted a profound impact on cellular functions, including cell movement and molecular binding. Since abnormalities in the WNT pathway have been reported in patients with malignant PCa, we then studied whether GIPC2 impacts PCa metastasis through Wnt signaling. To explore details of PCa metastasis and focus, we reanalyzed the different expression of genes using Cufflinks/Cuffdiff and applied both fold change (FC) (>2) and FDR 0.05 cut-off values to our gene list and detected several categories of tumor metastasis-associated genes. Core analysis and causal effects analysis in GIPC2-overexpression predicted abnormal functional associations with genes involved in cell motility, molecular binding, transport functions, and inflammatory processes. The IPA network and upstream analysis demonstrated that many of these effectors were involved in WNT-related pathways (Fig. 4r). Moreover, increased GIPC2 expression was linked to increased cellular movement and immune cell trafficking. Overlaying canonical pathways with molecular binding activity showed that several major cancer pathways were associated with genes involved in cellular movement.

### GIPC2 activated WNT-β-catenin signaling in PCa metastasis

**Fig. 5:**
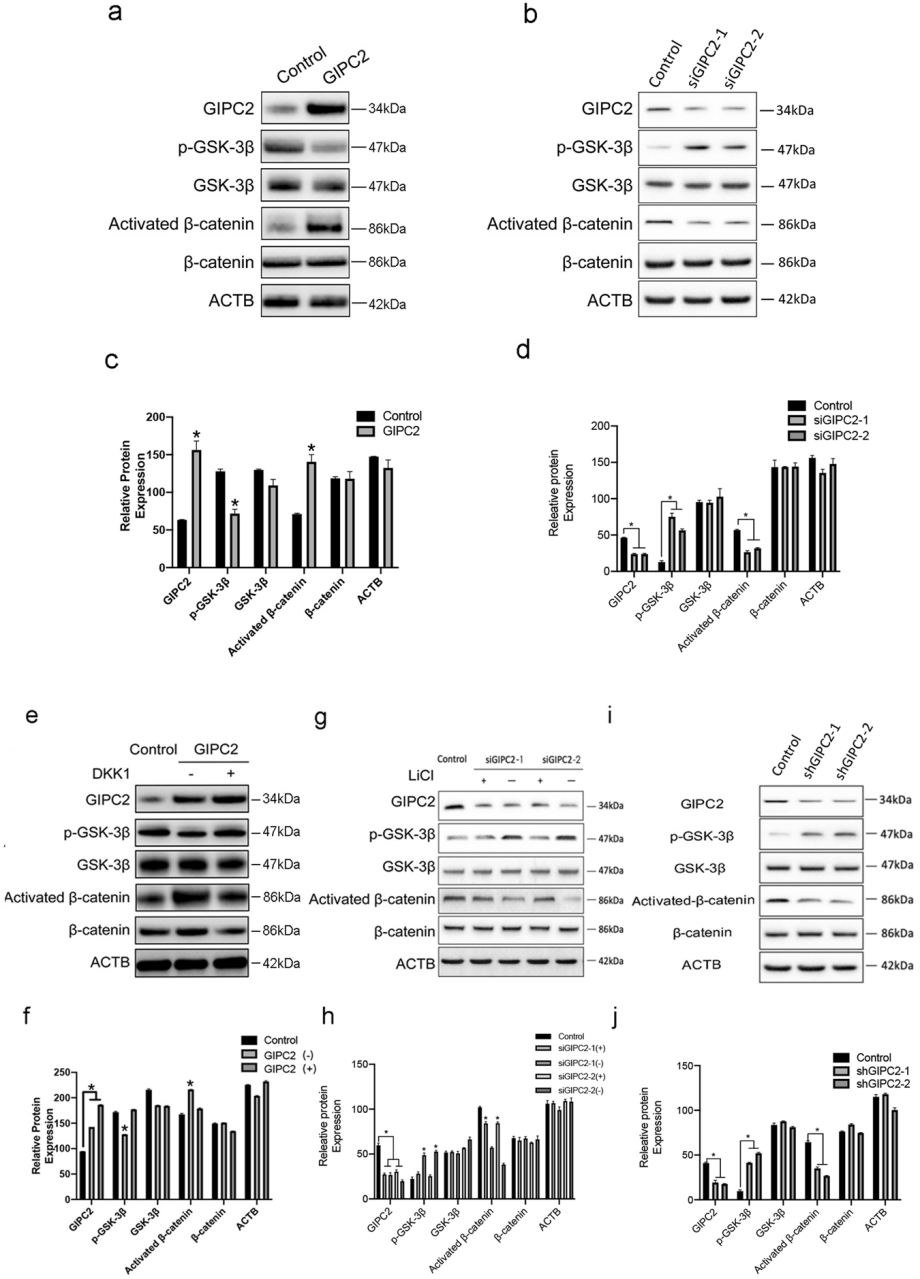
GIPC2 promoted WNT–β-catenin-pathway activation. **a**, **b** RWPE-1 and C4-2 cells were transfected with pcDNA-GIPC2 or siGIPC2-1/siGIPC2-2 for 48 h. Then, the protein-expression levels of GIPC2, p-GSK-3β, total GSK-3β, activated β-catenin, and total β-catenin were analyzed by western blotting, with ACTB serving as a loading control. **c**, **d** Graphical display of target protein-expression levels normalized to ACTB expression. **e**, **f** Treating RWPE-1 cells with 0.25 μg DKK1 (an inhibitor of the Wnt–β-catenin-signaling pathway), with or without GIPC2 overexpression. Total proteins were harvested at the indicated time points for western blot analysis. **g**, **h** C4-2 cells were treated with 5 mM LiCl (an activator of the Wnt–β-catenin-signaling pathway) for 24 h, following GIPC2 downregulation. Protein-expression levels of GIPC2, p-GSK-3β, total GSK-3β, activated β-catenin, and total β-catenin were analyzed by western blotting, with ACTB serving as a loading control. **i**, **j** In tumor tissue homogenates from mouse xenografts, protein expression levels of GIPC2, p-GSK-3β, and activated β-catenin were measured to verify the effects of GIPC2 knockdown. Western blot-band intensities were measured using Image J software. Normalization was done by dividing the target signal by the ACTB signal. P values were determined by Student’s *t* test. The results are presented as the mean ± SD. *p < 0.05, *n* = 3.

Next, we studied key molecules associated with the WNT-β-catenin pathway, including GSK-3β and β-catenin. GIPC2 overexpression in RWPE-1 significantly decreased (*p* < 0.05) phosphorylated (p)-GSK-3β and activated β-catenin expression, compared with controls (Fig. 5a, c). To further investigate the effects of GIPC2 loss in vitro, C4-2 cells were transfected with GIPC2 siRNA to knockdown GIPC2. The efficiency of the siRNAs was determined by western blotting. C4-2 cells treated with GIPC2 siRNA exhibited significantly higher p-GSK-3β expression and lower β-catenin activation compared with control siRNA- or mock-treated cells (Fig. 5b, d).

To confirm GIPC2 activated the WNT–β-catenin pathway, we used Dickkopf1 (DKK1) to inhibit the WNT signaling in RWPE-1 cells. DKK1 treatment abolished β-catenin accumulation stimulated by GIPC2 overexpression (Fig. 5e, f). Conversely, we used LiCl (a GSK-3β inhibitor) to activate WNT signaling in C4-2 cells. p-GSK-3β production and β-catenin activation were rescued in C4-2 cells by LiCl treatment compared with vehicle treated controls (Fig. 5g, h).

In tumor tissue homogenates from mouse xenografts, protein expression of GIPC2, p-GSK-3β, and activated β-catenin were measured to verify the effects of GIPC2. Compared with controls, p-GSK-3β was enhanced and activated β-catenin was reduced in the GIPC2-knockdown group (Fig. 5i, j).

### GIPC2 bound Fzd7 via the PDZ domain

**Fig. 6:**
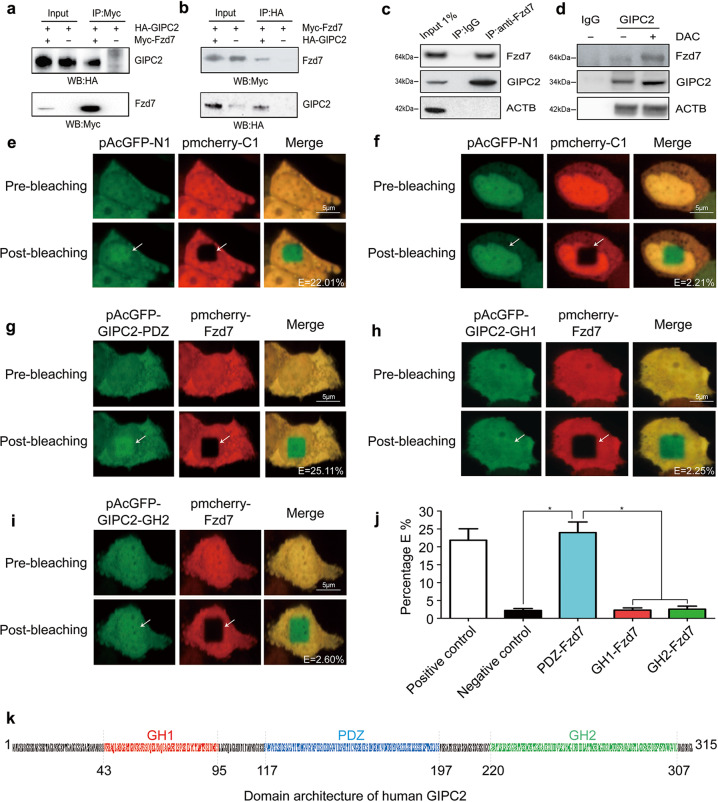
GIPC2 interacted with the PDZ domain of Fzd7. **a**, **b** Exogenous GIPC2 associated with exogenous Fzd7. Total lysates from C4-2 cells expressing GIPC2-HA and/or Fzd7-Myc were immunoprecipitated with antibodies against Myc (**a**) or the HA tag (**b**), followed by western blotting using the indicated antibodies. **c** Endogenous GIPC2 interacted with endogenous Fzd7. C4-2 cell lysates were immunoprecipitated with an anti-Fzd7 antibody, followed by western blotting using an anti-GIPC2 antibody. **d** C4-2 cells were treated with or without DAC. Endogenous GIPC2 from C4-2 cells was analyzed by western blotting with an anti-Fzd7 antibody or immunoprecipitated with an anti-GIPC2 antibody. **e** Positive FRET images. C4-2 cells, co-expressing pmCherry-Fzd7 and three different GFP–GIPC2 domains, were analyzed by confocal microscopy. Positive FRET image of C4-2 cells co-transfected with pairs of GFP-C1 and Red-N1 fusion crystallins. **f** Negative FRET images. Images were acquired before and after photobleaching. A nonbleached region and a corresponding bleached region (shown by an arrow) were used for the data analysis, compared with an unbleached cellular region as a negative control. **g** C4-2 cells co-transfected with pAcGFP-GIPC2-PDZ (green) and pmCherry-Fzd7 (red). **h** C4-2 cells co-transfected with pAcGFP-GIPC2-GH1 and pmCherry-Fzd7. **i** The images show C4-2 cells co-transfected with pAcGFP-GIPC2-GH2 and pmCherry-Fzd7. **j** FRET efficiencies (percentages) for the interaction between two crystallin partners were calculated, and the FRET intensity was analyzed statistically. The FRET intensity expressed as a percent represents the fraction of interacting donor molecules. The results suggested that a significant interaction occurred between GIPC2-PDZ and Red-Fzd7. **k** Domain architecture of GIPC2. The GIPC2 protein consists of a GH1 domain (red), a PDZ domain (blue), and a GH2 domain (green). The amino acid position is shown on the domain architecture. The GH1 domain in the N-terminal region, the PDZ domain in the middle region, and the GH2 domain in the C-terminal region are well conserved among GIPC family members.

Next, we used MS to screen for molecules that could directly bind GIPC2 to gain insight into its potential role as an oncoprotein. HA-GIPC2 was precipitated from RWPE-1 cells after in-gel trypsin digestion and Linear ion trap-Fourier transform ion cyclotron resonance mass spectrometry (LTQ-FT-MS) identification. We identified candidate proteins that could physically interact with GIPC2 (Supplementary Table S5). We hypothesized that GIPC2 may interact with Fzd7, which exerts an oncogenic role in WNT-signal activation. To test this hypothesis, we co-expressed Myc-Fzd7 and GIPC2-HA in RWPE-1 cells, and Fzd7 and GIPC2 were immunoprecipitated with an anti-Myc (Fig. 6a) or anti-HA antibody (Fig. 6b), followed by western blotting. Reciprocal co-IP experiments showed that Myc-Fzd7 interacted with GIPC2-HA. Endogenous GIPC2 also immunoprecipitated with the Fzd7 antibody (Fig. 6c). The GIPC2–Fzd7 interaction was further confirmed by DAC treatment in RWPE-1 cells. In the presence of DAC, detectable endogenous GIPC2 was pulled down by the GIPC2 antibody (Fig. 6d). Our results indicated that GIPC2 directly interacted with Fzd7 in PCa cells.

GIPC2 contains a GH1 domain, a PDZ domain, and a GH2 domain that are conserved in the GIPC family (Fig. 6k). These domains were individually conjugated with pAcGFP (green), and Fzd7 was tagged with a red fluorescent dye (pmCherry). Fluorescence resonance energy transfer (FRET) was used to provide direct visual evidence of protein–protein interactions. FRET-acceptor photobleaching involves distance-dependent (20–60 Å) interactions between excited fluorescent dye molecules from donor molecules to acceptor molecules, without exciting photons. E% values were calculated as the ratio of GFP fluorescence before (pre-GFP) photobleaching and after (post-GFP) photobleaching. GFP and pmCherry images were recorded before and after acceptor photobleaching. As a positive control, a GFP-pmCherry fusion protein separated by 10-amino acid linkers (C1 and N1) was used. The ROI in cells expressing fusion proteins was photobleached to 30% of the original intensity. Cells transfected with the positive control showed an average FRET efficiency (E%) of 22.01% after photobleaching (Fig. 6e, j) versus the negative control (Fig. 6f, j). Negative-control cells transfected with unlinked GFP and pmCherry constructs showed no changes in GFP fluorescence after regional bleaching (E value=2.21%; Fig. 6f, j). C4-2 cells co-transfected with pAcGFP-GIPC2-PDZ and pmcherry-Fzd7 showed an average E value of 25.11%, which was higher than that of the positive control after photobleaching (Fig. 6g, j, Supplementary Table S6). We observed acquired fluorescence after photobleaching in C4-2 cells co-transfected with pmcherry-Fzd7 and pAcGFP-GIPC2-GH1 or pAcGFP-GIPC2-GH2 (Fig. 6h, i), with average E-values of 2.25–2.60%, similar to negative-control cells (Fig. 6f).

### The GIPC2–PDZ–Fzd7 axis promoted PCa metastasis through the WNT–β-catenin pathway

**Fig. 7:**
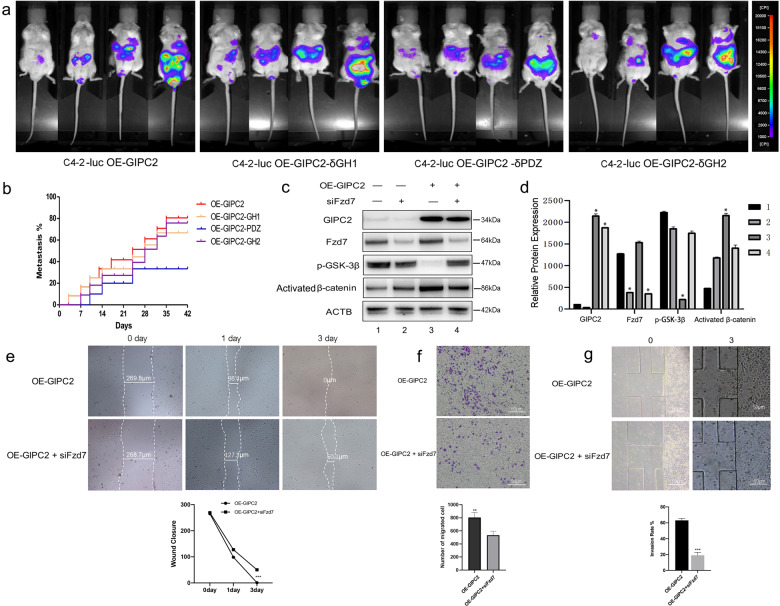
The GIPC2–PDZ–Fzd7 axis activated the WNT–β-catenin pathway in prostate cancer metastasis. **a** Luciferase-expressing C4-2 cells (2 × 10^5^) were suspended in 15 μL of sterile PBS and used for intracardiac injections of male BALB/c nu/nu mice with different GIPC2 isoforms (wild-type GIPC2, GIPC2-δGH1, GIPC2-δGH2, and GIPC2-δPDZ, *n* = 8 of each group). Bioluminescent images of metastatic tumors were monitored. Each picture includes four different time points: 7, 14, 21, and 28 days. The colored scale bars represent low (purple) to high (red) tumor burdens. **b** The metastasis rate was determined using the Kaplan–Meier method. *p < 0.05 between GIPC2-δPDZ group and three other groups. **c** Western blotting showed GIPC2, Fzd7, p-GSK-3β, and activated β-catenin expression in C4-2 cells transfected with OE-GIPC2 (GIPC2 overexpression) or si-Fzd7. **d** Graphical display of target protein-expression levels normalized to ACTB expression. **e** Cell-migration abilities were measured by performing wound-healing assays. C4-2 cells were transfected with pcDNA-GIPC2 (with or without si-Fzd7) to evaluate the pro-migration and pro-invasion effects of the GIPC2–Fzd7 axis. After scratching a wound and removing the floating cells, prostate cancer cells (untreated or control-treated) were used in wound-scratch assays for 0, 1, or 3 days. **f** Prostate cancer cells treated as described above were applied to transwell chambers coated with Matrigel and incubated for 24 h. **g** The microfluidic model consists of two independent microchannels, where tumor cells and 20% FBS were seeded. Between the two channels, 3D Matrigel was seeded to mimic the ECM. Invading cells was observed under a light microscope (200×). The data shown represent the mean ± SD. *p < 0.05; **p < 0.01.

To confirm that GIPC2**–**Fzd7 binding facilitated PCa metastasis, we investigated different GIPC2 isoforms. We individually overexpressed the GIPC2-δGH1 (GH1 deletion), GIPC2-δGH2 (GH2 deletion), or GIPC2-δPDZ (PDZ deletion) isoforms. The GIPC2-δGH1 and GIPC2-δGH2 had similar effects on PCa metastasis compared with wild-type GIPC2 (Fig. 7a). However, deleting the PDZ domain distinctly influenced cell motility in vivo. Integrated luminescence values of the ROI were used for statistical analyses of tumor growth and metastasis. PCa growth was not altered by GIPC2-GH1 and GH2 deletion (Fig. 7a). Furthermore, tumor metastasis was increased by GIPC2 overexpression, but not by GIPC2-PDZ deletion (Fig. 7a, b).

While exploring the role of the GIPC2–PDZ–Fzd7 axis in the WNT-signaling pathway in PCa metastasis, we found that p-GSK-3β decreased and that activated β-catenin increased in Fzd-knockdown PCa cells (Fig. 7c, d). Moreover, the metastasis-promoting effect of GIPC2 overexpression was abolished by Fzd7 downregulation (Fig. 7e–g). The GIPC2–PDZ–Fzd7 axis affected WNT levels through p-GSK-3β production and β-catenin activation (Fig. 7c). GIPC2 facilitated WNT–β-catenin-pathway activation through GIPC2 binding with the Fzd7 PDZ domain.

### Exosome-derived GIPC2 may serve as a marker of mPCa progression and diagnosis

**Fig. 8:**
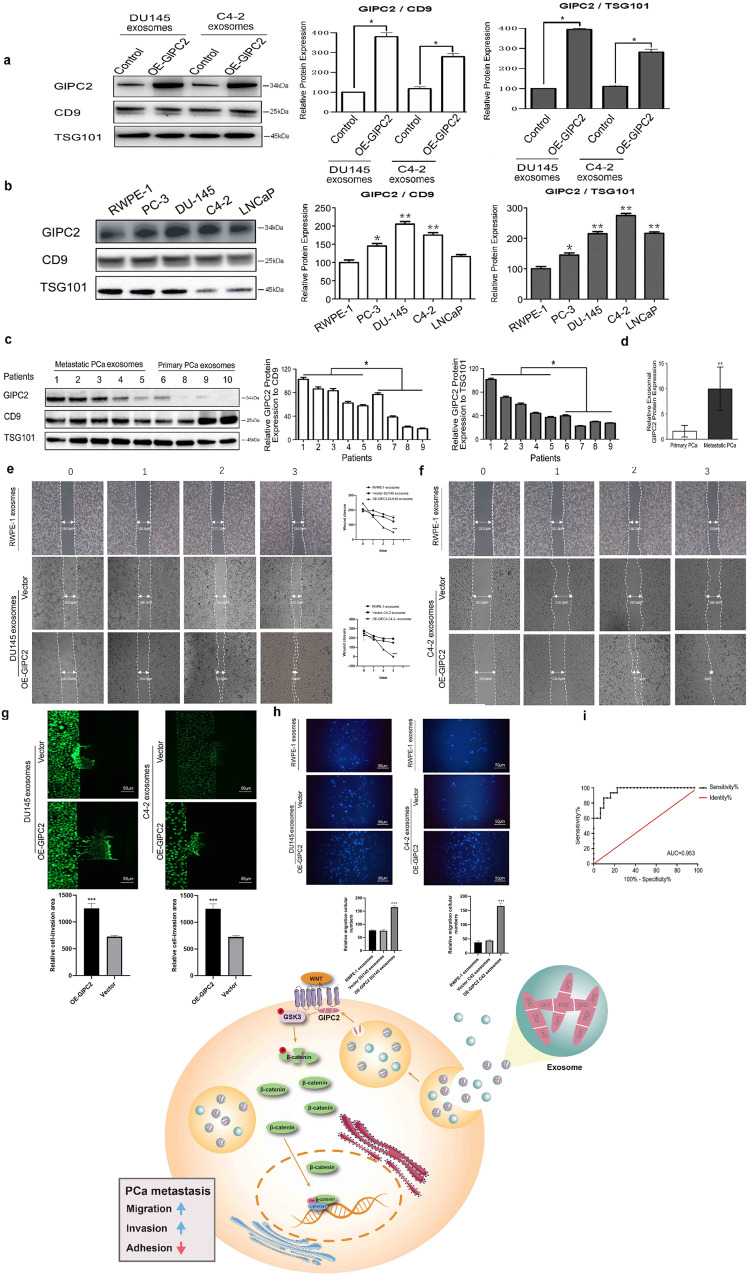
GIPC2 derived from exosomes can potentially be considered for metastatic prostate cancer diagnosis. **a**, **b** Exosomes were isolated from the supernatants of prostate cancer cell lines. Exosome-marker proteins (including CD9 and TSG101) were analyzed by western blotting. GIPC2 was also observed in supernatants and exosomes from prostate cancer cell lines. **c**, **d** The characteristics of exosomes from primary prostate cancer samples and metastatic prostate cancer patients were investigated by western blotting, based on GIPC2 expression and the exosome-specific expression of CD9 and TSG101. **e**, **f** Cell-migration abilities were measured by performing wound-healing assays. Treating RWPE-1 cells with exosomes isolated from RWPE-1, GIPC2-overexpression or empty vector-overexpression tumor cells (C4-2 and DU145) to investigate pro-migration and pro-invasion effects. After scratching a wound and removing the floating cells, RWPE-1 cells (untreated or control-treated) were used in wound-scratch assays for 0–3 days. **g**, **h** Cell invasion was analyzed in transwell assays and with the microfluidic platform. C4-2 treated as described above were applied to transwell chambers coated with Matrigel and incubated for 24 h. The microfluidic model consists of two independent microchannels, where tumor cells and 20% FBS were seeded. Between the two channels, 3D Matrigel was seeded to mimic the ECM. Invading cells were labeled with Calcein-AM (green). The data shown represent the mean ± SD. *p < 0.05; **p < 0.01. **i** ROC-curve analysis for GIPC2 showed higher AUC values for distinguishing between primary or metastatic prostate cancer (**p < 0.01, ***p < 0.0001, two-sided Mann–Whitney test). **j** Schematic representation of the mechanism whereby GIPC2 promotes tumorigenesis in prostate cancer. The GIPC2–PDZ–Fzd7 pathway in prostate cancer is shown.

Exosomes derived from mPCa were detected in urine-accumulated RNA and proteins in a kind of pre-metastatic niche, indicating the vital functions of exosomes in cancer metastasis [29, 30]. The GIPC2 structure indicated a probable membrane localization in exosomes [19]. Herein, we identified GIPC2 in exosomes derived from PCa cells (Fig. 8a, b). When equal protein quantities were isolated from exosomes, western blotting analysis revealed GIPC2 was highly expressed in exosomes derived from metastatic, but not primary, PCa samples (Fig. 8c, d).

Next, we tested whether tumor-derived, GIPC2-positive exosome treatment affected PCa cell motility. RWPE-1 cells were treated for 24 h with exosomes isolated from RWPE-1, C4-2, or DU145 transfectant-conditioned media. Increased RWPE-1 cell adhesion, invasion, and migration were observed when cells were treated with exosomes from GIPC2-high expression tumor cells versus control exosomes (Fig. 8e–h). The importance of exosomal GIPC2 was confirmed using exosomes produced by C4-2 cells. Treating RWPE-1 cells with C4-2 cell-derived, GIPC2-positive exosomes strongly and positively influenced cell motility, comparable to that observed with GIPC2 overexpression.

Western blotting measured normalized, urinary GIPC2 protein levels in samples from our cohort of 46 patients (31 primary and 15 metastatic tumors from bones). The area-under-the-curve (AUC) values for GIPC2 (Fig. 8i) were 0.953 (95% confidence interval [CI], 0.75–0.95; sensitivity, 83.3%; specificity, 83.3%) and 0.80 (95% CI, 0.899–1.007; sensitivity, 86.7%; specificity, 90.3%) for the primary and metastatic tumors, respectively.

## Discussion

We describe a novel conditional oncogene, GIPC2 located on chromosome 1p31.1, whose activation was associated with abnormal promoter methylation in PCa. We also provide evidence of oncogenic effects on PCa metastasis in vitro and in vivo by regulating GIPC2 expression. This is the first study indicating that GIPC2 promotes tumor metastasis independently of cell proliferation and apoptosis through the WNT–β-catenin pathway, and is thus promising as a novel drug target. As we know, the WNT–β-catenin pathway plays a key role in a subpopulation of PCa patients with metastasis [12, 14]. However, previous studies have provided contradictory evidence of canonical WNT/β-catenin signaling in PCa [14, 17]. Molecular studies investigating PCa mortality and metastatic progression have suggested that the Akt–PI3K-pathway activation phosphorylates GSK-3α and/or inhibits GSK-3β activity [31–33]. However, the mechanism of WNT–β-catenin-pathway activation in PCa metastasis remains unknown [34]. GIPC2 expression may stimulate PCa metastasis (Fig. 4) by binding to the WNT co-receptor Fzd7 (Fig. 5) [13]. Evidence for increased expression of WNT co-receptors in PCa is not as prevalent as for Fzd-family members (including Fzd 1, 2, 5, 7, and 8), which are overexpressed in PCa [35]. Preclinical results support upregulated Fzd7 as a promising target in PCa [36, 37], but Fzd7 showed invariable expression in our collected PCa samples (Fig. S9). We conclude that Fzd7 mediated WNT–β-catenin-pathway activation via GIPC2 overexpression in mPCa. Our findings are promising for a subset of patients with aggressive PCa in clinical trials evaluating WNT inhibitors, particularly for agents targeting WNT secretion and/or WNT receptor binding, and those preventing Fzd-family interaction with key factors, such as GIPC2 (Fig. 8j). We propose a GIPC2-centered unified model for the development of metastasis in PCa that does not exclude the participation of other coordination genes in the oncogenic transformation.

Molecules involving PDZ presumably reflect fundamental differences in signaling networks between cell types. Indeed, our observation of GIPC2 abnormal methylation as well as higher GIPC2 expression in mPCa suggests that GIPC2 activation rather than inactivation is favorable for cancer formation. As a paralogous gene in the GIPC family, GIPC2 has a highly conserved PDZ domain, which is often considered an adapter region mediating protein–protein interactions [19]. The PDZ domain in the GIPC family interacts with various proteins, including transmembrane proteins, kinases, glycoprotein, and viral proteins. In pheochromocytoma/paraganglioma (PPGL), we previously demonstrated that GIPC2 is a tumor suppressor with loss of function triggering tumorigenesis via loss of heterogynous at GIPC2 locus and hypermethylation of GIPC2. The PDZ domain of GIPC2 physically interacted with nucleoprotein NONO to inactivate p27 which promoted oncogenic transformations leading to PPGL [28]. In PCa cells, we identified a novel function for GIPC2 as a PCa oncogene which is opposite to that of in PPGL. Further, we demonstrated that GIPC2 directly binds to Fzd7 via the PDZ domain and behaved similarly to the disheveled homologs (DVL) protein in WNT–β-catenin-pathway activation in mPCa (Figs. 5, 6). Indeed, in PCa, GIPC1 as a paralogous gene of GIPC2—which is involved in endosomal signaling and tumor cell proliferation, invasion, and metastasis [19, 38]—the PDZ domain of DVL binds the C-terminal region of Fzd receptors and is essential for WNT-signal transduction in PCa tumorigenesis [39]. Moreover, several DVL inhibitors can inhibit proliferation of the PCa PC3 cells. Thus, we speculate that GIPC2 is a novel oncogene that disrupts the destruction complex, whose components include axin, glycogen synthase kinase-3 (GSK-3), casein kinase 1, and adenomatous polyposis coli protein (APC), resulting in β-catenin stabilization, translocation, and degradation [12, 14, 40]. Further studies should focus on identifying ligands of the PZD domain in GIPC2 for different cancer types. Given our findings and the established role of GIPC2 as an oncogene in the prostate, we propose that GIPC2 function may be context-dependent.

In summary, we identified GIPC2 as a novel oncogene in PCa and provided evidence that it can promote metastasis-independent growth in PCa cells. The nature of the oncogenic effect of GIPC2 was clearly shown by the reconstitution and knockdown experiments. Moreover, using a microfluidic array, we demonstrated that GIPC2 deficiency dramatically restricted tumor cell invasion and migration, accompanied by WNT-β-catenin-pathway activation. In contrast to the role of GIPC2 as a putative tumor suppressor in ALL and PPGLs [28], [24], the potential oncogenic function of GIPC2 in PCa demonstrated a cell-type dependent function. By addressing the distinct functions of GIPC2 in PCa progression and metastasis, our results strengthen the understanding of GIPC2-linked epigenetic alterations in tumorigenesis and as a potential chemotherapeutic target associated with WNT signaling. Our findings lay a foundation for further investigations on the role of GIPC2 in tumors and provides evidence supporting epigenetic therapy in treating mPCa.

## Material and methods

### Patients and samples

Primary tumors (*n* = 36) or metastatic prostate tumors (*n* = 17), and matching normal adjacent tissues, were derived from radical prostatectomy of 53 PCa patients [27]. The clinical parameters of all PCa patients are presented in Supplementary Table S1. mPCa tumors were confirmed by distant metastatic neoplasms from bones and tumor pathologies were verified by a uropathologist. The collection and use of PCa tissue samples were reviewed and approved by the Institutional Ethics Committee of the First Affiliated Hospital of Dalian Medical University (China). Informed consent for publishing data relating to individual participants was obtained from the participants (or legal guardian).

The patient cohort was assembled previously [27]. Fifty-three patients treated in The First Affiliated Hospital of Dalian Medical University (Dalian, China) were included in this study (Supplementary Table S1). Seventeen patients with evidence of mPCa were monitored with follow-up investigations. All specimens were analyzed according to our previous study [27]. Prostate tissue specimens were treated overnight with RNALater at 4 °C according to the manufacturer’s instructions and then stored at −80 °C.

### RNA sequencing and data analysis

We compared differences in gene expression between localized tumors and metastatic tumors. RNAseq data from patients with primary or mPCa (*n* = 20) was utilized to assess gene expression. Patients with *de novo* metastasis (clinical stage M1, *n* = 10) and localized (clinical stage P0, *n* = 10) tumors were enrolled from the First Affiliated Hospital of Dalian Medical University. In total, 100 ng total RNA was used to generate the libraries according to manufacturer’s instructions (TruSeq RNA Access Library Prep Kit, Illumina). Library quality control was performed with the Qubit 4.0 and bioanalyzer (Agilent), followed by Nexseq550 sequencing and analysis of data at PE50. For RNAseq data analysis, a filtered dataset was quantified according to gene-level expression from RNAseq results performed using build GRCh38/hg38 as the Homo Sapiens reference genome. Differentially expressed genes (DEGs) were selected with a *p* value < 0.01, FDR < 0.05 and FC ≥2, to determine whether a set of genes showed statistically significant and/or concordant differences between two biological states such as M1 versus P0. For the training and evaluation of the classifier using the gene signatures, two principal dimensions using principal component analysis were extracted from expression matrix of the gene signature and then SVM algorithm was applied to determine the discrimination border between the two groups (M1 versus P0).

### Re-analysis of the public dataset of primary PCa and mPCa

RNASeq data (read counts per gene evaluated by featureCounts) and mRNA-expression array files including 246 mPCa and 55 primary PCa were downloaded from the TCGA (phs000178) and GEO database (GSE147250), and were then analyzed in the statistical environment using R. Data normalization and gene expression analysis was performed using the DESeq2 package. The obtained results were considered statistically significant when the p-adj value was <0.05. Illumina Casava v1.7 software was used for basecalling. The quality of sequence reads from the RNAseq data were assessed and low-quality reads were filtered using the FastQC tool (Babraham Bioinformatics, Cambridge, UK) and ShortRead (v.1.30.0) package from R bioconductor (v3.3). Quantification of gene-level expression from preprocessed RNAseq results were performed using the UCSC hg19 build of the Homo Sapiens genome, using the Subread aligner and featureCounts software.

### Cell culture

Prostate tumor cell lines (American Tissue Culture Collection) were maintained according to manufacturer’s guidelines. C4-2 and DU145 cell lines were cultured in RPMI-1640 media (72400047, Gibco) containing 10% FBS (16140071, Gibco) and 1% penicillin–streptomycin (10378016, Gibco). RWPE-1 cells were maintained in Keratinocyte-SFM medium (10744019, Gibco). All cells were cultured in a humidified incubator (Heracell 2401, Thermo Fisher) at 37 °C with 5% CO_2_ [27].

### Protein extraction and western blot analysis

Total cellular proteins were extracted using M-PER™ Mammalian Protein Extraction Reagent (78501, Thermo) and phenylmethanesulfonyl fluoride. Lysate protein concentrations were quantified with a Bicinchoninic Acid Protein Assay Kit (P8340, Thermo) Twenty micrograms of the protein was separated by 4–12% SDS-PAGE gel and transferred to polyvinylidene fluoride (PVDF) membranes (IPVH00010, Millipore). Before antibody incubation, PVDF membranes were blocked with 5% non-fat milk solution in TBS with 0.05% Tween-20 at room temperature for 1 h. The membranes were hybridized individually overnight at 4 °C with antibodies in the Cell Cycle Regulation Antibody Sampler Kit (Cell Signaling Technology) and those recognizing GIPC2 (ab175272, Abcam), β-catenin (ab32572, Abcam), Fzd7 (ab64636, Abcam), and ACTB (3700T, Cell Signaling Technology). The blots were incubated with a horseradish peroxidase (HRP)-conjugated secondary antibody (Millipore) for 1 h at room temperature and detected by Super Signal West Pico Chemiluminescent Substrate (Pierce) using an LAS-3000 mini-imaging system (Fuji Film). The signal intensity of each antibody was quantified using Image J software. All results are presented as the mean ± SD (*p < 0.05, *n* = 3).

### Quantitative real-time reverse transcription (RT)-PCR

Cells were harvested and total RNA was extracted. cDNAs were synthesized by M-MLV reverse transcriptase (M1701, Promega) and analyzed by real-time PCR using an ABI 7500 Real-Time PCR System (Applied Biosystems). Amplification was conducted as follows: 95 °C for 10 s followed by 40 cycles of 95 °C (5 s) and 60 °C (34 s). Each sample was tested in triplicate, and the PCR products were verified by DNA sequencing. Results from three independent experiments were used to calculate relative gene-expression levels, using the 2−ΔΔCT method and mRNA-expression levels were normalized to that of ACTB.

### Immunohistochemistry

Paraffin sections (8 μm) were stained with antibodies against GIPC2 (sc-515441, Santa Cruz) or BMI-1 (ab126783, Abcam) at a 1:100 dilution. The sections were incubated with biotinylated IgG (1:200, ZSGB-Bio Co.) at 37 °C for 20 min, followed by incubation with HRP-labeled streptavidin at 37 °C for 15 min. Color development proceeded for 5 min at room temperature using diaminobenzidine, followed by rinsing with distilled water. Counterstaining was performed with hematoxylin. The immunohistochemistry (IHC)-staining intensity was classified based on the quantized values from Image J, as follows: +1, <1.00 positive staining; +2, 1.01–5.00 positive staining; +3, >5.01 positive staining.

### Microfluidic chip fabrication

Previously developed invasion and metastasis microfluidic devices [41, 42] were employed. A monolayer-invasion microfluidic chip and a multilayer-metastasis microfluidic chip were constructed of poly-dimethylsiloxane (PDMS, Silgard 184, Dow Chemical), which was replicated from SU-8 3035 negative photoresist (Microchem Corp.)-patterned wafers. Cell-inlet holes and culture chambers were created using differently sized punchers (1.5 mm and 3 mm diameter). After the PDMS chips were prepared, they were bonded irreversibly to a glass substrate after oxygen-plasma treatment for 90 s. Before use, the device was sterilized with UV light for 30 min.

### Cell-adhesion assays with the microfluidic devices

An adhesion microfluidic device was previously confirmed as an ideal model for mimicking circulating tumor cell adhesion [43, 44]. To mimic the endothelial barrier of blood vessels, human umbilical vein endothelial cells (HUVEC) were seeded in the microchannels; HUVEC cells attached to the porous membrane and formed a monolayer barrier, to mimic the barrier in vivo. The bottom chambers were loaded into 100 ng/mL EGF and incubated for 2 h to stimulate circulating tumor cell adhesion. Meanwhile, the experimental tumor cells were labeled with Calcein-AM. Next, the syringe pump regulated the flow of tumor cells at 750 nL/min for 30 min to achieve a steady state in the microchannels. Then, the pump was stopped, and the tumor cells were incubated for another 10 min and PBS was used to remove any loosely attached cells. The adherence ability of the tumor was directly proportional to the intensity of green fluorescence signal, which reflected the number of residual tumor cells. Images of each microchannel were recorded with a fluorescence inversion microscope (Leica).

### Cell-invasion and migration assays

Invasion and migration assays were performed in Transwell plates (BD Biosciences) according to the manufacturer’s protocol. Briefly, cells were plated on Matrigel (BD Biosciences)-coated chambers of 24-well plates at 5 × 10^4^ cells/well in medium without serum. Culture medium (800 µL) containing 10% FBS was added to the bottom chambers. After a 24-h incubation, the lower side of each Transwell membrane was fixed and stained with 0.05% crystal violet. The filters were observed under an inverted microscope (Olympus). The cells in each well in three random microscopic fields/filter were counted at ×200 magnification. Data are presented from three independent experiments, performed in duplicate.

Next, The reconstitution of the microfluidic model according to the previous study [45]. The invasion microfluidic chip consisted of 3D-culture units (~50 μm) and two side medium channels (~100 μm) that mimic the process of tumor cell invasion into the extracellular matrix. Matrigel, which mimics the extracellular matrix, was loaded into the 3D-culture units. Tumor cells (8 × 10^3^) in serum-free medium were seeded into one side of the medium channel. After a 14-h culture, highly concentrated FBS or growth factors were injected into the other side. Each chip was incubated for 48 h at 37 °C, 5% CO_2_ and fluorescence-microscopy images were collected every 2 h. The cell-invasion area in the Matrigel served as quantifiable measure of invasion.

### Microarrays and ingenuity pathways analysis (IPA)

Gene-expression-profile data were analyzed using RNA from RWPE-1 cells overexpressing GIPC2 and control cells on the Affymetrix HTA-2.0 microarray platform. Sample preparation, hybridization, and image acquisition were performed as per the manufacturer’s instructions. The raw data were processed as described previously [27]. Expression-profile data obtained from one-way ANOVA were further analyzed. Genes were selected that were either induced (FC > 2) in treated cells or repressed (FC < −2) in control cells. The gene set was imported into IPA software. Gene networks representing key genes were identified using the curated IPA Knowledgebase.

### Mouse xenograft studies

Roughly equal numbers of male mice were randomly assigned to each experimental group. Before experiments were performed, it was established that only animals with normal phenotypes would be included in the study. To generate metastases, groups of eight male nude mice (strain BALB/c nu/nu, 4–5-weeks old), received intracardiac injections of 2 × 10^5^ C4-2/DU145-luc cells overexpressing empty vector or shRNA (Origene, USA) in 100 μL sterile PBS. Tumor cells were injected into the left cardiac ventricle. Mice were injected with 120 mg/kg luciferin, and metastatic dissemination of the cells was monitored using an IVIS Lumina II imaging system (Caliper LifeSciences) at regular intervals. Metastatic burden was quantified using the Living Image software (Caliper Life Sciences) by measuring the luminescent signal from each region of interest (ROI).

The mice were euthanized by CO_2_ asphyxiation 3–4 weeks after tumor cell injection, the organs were removed, and the metastatic burden of different organs was quantified using the IVIS Lumina II device. A subset of the collected tissues was fixed in formalin, embedded in paraffin and sectioned to 8-μm slices onto charged glass slides. Paraffin sections were stained with hematoxylin and eosin (H&E). During data analysis, investigators were blinded to specific treatment groups. All experiments were approved by Dalian Medical University Committee on Animal Resources.

### Exosome isolation

FBS was filtered using a 100-nm filter, then ultracentrifuged for 16 h, and then filtered again using a 100-nm filter. The supernatant was collected from cells that were cultured in media containing exosome-depleted FBS for 48 h, and was subsequently subjected to sequential centrifugation steps at 800 × *g* for 5 min, and 2000 × *g* for 10 min. This resulting supernatant was then filtered using 0.2-μm filter, and a pellet was recovered at 100,000 × g in a SW32 Ti rotor after 2 h of ultracentrifugation (Beckman). The supernatant was removed by aspiration and the pellet was resuspended in PBS and subsequently ultracentrifuged at 100,000 × *g* for an additional 2 h. The purified exosomes were then analyzed and used for experimental procedures. For the treatment of exosomes with proteinase K, purified exosomes were incubated (37 °C, 30 min) with 5 mg/mL proteinase K (Sigma-Aldrich, dissolved in RNase-free water) followed by heat inactivation (60 °C, 20 min). For RNase treatment, purified exosomes were incubated (37 °C, 30 min) with 2 mg/mL of protease-free RNase A (Thermo Scientific) followed by addition of 10X concentrated RNase inhibitor (Ambion).

### Statistical analysis

All data are presented as the mean ± standard deviation (SD). Statistical analysis and graphical data representation were performed using GraphPad Prism 5.0 (GraphPad Software, San Diego, CA, USA). Statistical significance was evaluated using Student’s *t* tests, ANOVA, or *χ*^2^ tests, as appropriate. All experiments were performed in triplicate and the data met the assumptions of the statistical analysis.

## Supplementary information

Supplementary Figure Legends

Supplementary Figure S1

Supplementary Figure S2–S5

Supplementary Figure S6

Supplementary Figure S7

Supplementary Figure S8

Supplementary Figure S9

Supplementary Table S1

Supplementary Table S2

Supplementary Table S3

Supplementary Table S4

Supplementary Table S5

Supplementary Table S6
