# Supplementary material for: GIPC2 interacts with Fzd7 to promote prostate cancer metastasis by activating WNT signaling
Source: Oncogene. 2022 Mar 28;41(18):2609–23. doi: 10.1038/s41388-022-02255-4 (PMC9054671; doi:10.1038/s41388-022-02255-4)
Supplement: Supplementary file 1 — Supplementary Figure Legends [file 41388_2022_2255_MOESM1_ESM.docx]

**Supplementary Figure Legends**

**Figure S1.**

The mRNA-expression and protein-expression levels in DU145 transfected with control and siRNA of GIPC2 (siGIPC2-1/siGIPC2-2) and verification of the specificity of the GIPC2 antibody. (a)RT-PCR was performed with DU145 transfected with control and siGIPC2-1/siGIPC2-2. (b) Western blot was performed with DU145 transfected with control and siGIPC2-1/siGIPC2-2. Western blot-band intensities were measured using Image J software. (c) Xenograft + / - GIPC2 tumor samples were used to verify the specificity of the GIPC2 antibody.

**Figures S2–S5.**

Correlations between GIPC2 expression and clinical parameters of patients with prostate cancer.

GIPC2 expression correlated with the Gleason score (Figure S2), patient ages (Figure S3), pre-op TPSA (Figure S4), and pre-op F/TPSA (Figure S5). Real-time RT-PCR was performed with GIPC2 mRNA isolated from 36 primary prostate cancer samples, including 17 metastatic tumors and matched normal adjacent tissues. All measured expression levels were normalized to ACTB expression, and relative expression levels were analyzed. All experiments were repeated three times (***p* < 0.01, ****p* < 0.0001, two-sided Mann–Whitney test).

**Figure S6.**

GIPC2 is upregulated in metastatic than primary prostate cancer in public data from GEO (GSE147493). Volcano plot of distribution of differentially expressed genes between primary tumors and metastatic tumors. The dotted line in grey represents the cut-off, a measurement of gene expression fold-change on the X-axis versus a measure of statistical significance [−Log10 (P-value)] on the Y-axis. Differentially expressed genes are established at |log2(Fold change)| ≥0.586 and P-value < 0.05 (Blue and Pink dots).

**Figure S7.**

Re-analysis of GIPC2 expression in GEO and TCGA datasets. RNA-Seq and expression data (read counts per gene evaluated by feature Counts) including 246 metastatic PCa and 55 primary PCa were downloaded from the GEO(GSE147250) and TCGA database (phs000178), and were then re-analyzed in the statistical environment using R. Data normalization and gene expression analysis was performed using the DESeq2 package. The re-analysis results showed that GIPC2 expression in metastatic PCa were significantly higher than in primary PCa samples (****p* < 0.0001, nonparametric tests).

**Figure S8.**

The protein-expression levels were analyzed by western blotting. (a) C4-2 cells were transfected with control or or siRNA of GIPC2 (siGIPC2-1/siGIPC2-2). (b) RWPE-1 cells were transfected with control or pcDNA3.1-GIPC2. (c)Tumor part scraped from paraffin sections for H&E staining with DU145-luc Control shRNA or antoher shRNA of GIPC2 (shRNA-GIPC2-2) treatment in Fig.4q. (d) Tumor parts scraped from paraffin sections for H&E staining with C4-2-luc Control shRNA or shRNA-GIPC2-2 treatment in Fig.4q. Western blot-band intensities were measured using Image J software. Normalization was done by dividing the target signal by the ACTB signal. P values were determined by Student’s t test. The results are presented as the mean ± SD. ****p* < 0.001, n=3. (e) C4-2-luc Control or shRNA-GIPC2-2 (2 × 10^5^) and DU145-luc Control or shRNA-GIPC2-2 (2 × 10^5^) were suspended in 15 μL sterile PBS and used for intracardiac injections into male BALB/c nu/nu mice (n=8 of each group, 32 mice in all). Bioluminescent images of metastatic tumors were monitored. Metastasis rates were determined according to the Kaplan–Meier method. **p* < 0.05 between two groups. (f) Paraffin sections of organs were stained with hematoxylin and eosin (H&E) and the tumor tissue is indicated by an arrow. (g-h) The metastatic burden of different organs was quantified by monitored using an IVIS Lumina II (n=8 of each group). Colored scale bars represent low (purple) to high (red) tumor burdens.

**Figure S9.**

Fzd7 expression in prostate cancer samples. RT-PCR was performed with Fzd7 mRNA isolated from 36 primary prostate cancer tissues (including matched, normal adjacent tissues) and 17 metastatic tumor tissues. Fzd7 showed invariable expression in prostate cancer samples.

**Table S1.**

Clinical parameters of prostate cancer samples.

**Table S2.**

Summary of clinical PCa samples.

**Table S3.**

Database of the GEO (GSE147250) and TCGA (phs000178)

**Table S4.**

Different expession Gene list.

**Table S5.**

Potential interaction protein with GIPC2 by MS.

**Table S6.**

The average FRET efficiency (E) after photobleaching.
