# Supplementary figures and images for: GIPC2 interacts with Fzd7 to promote prostate cancer metastasis by activating WNT signaling
Source: Oncogene. 2022 Mar 28;41(18):2609–23. doi: 10.1038/s41388-022-02255-4 (PMC9054671; doi:10.1038/s41388-022-02255-4)

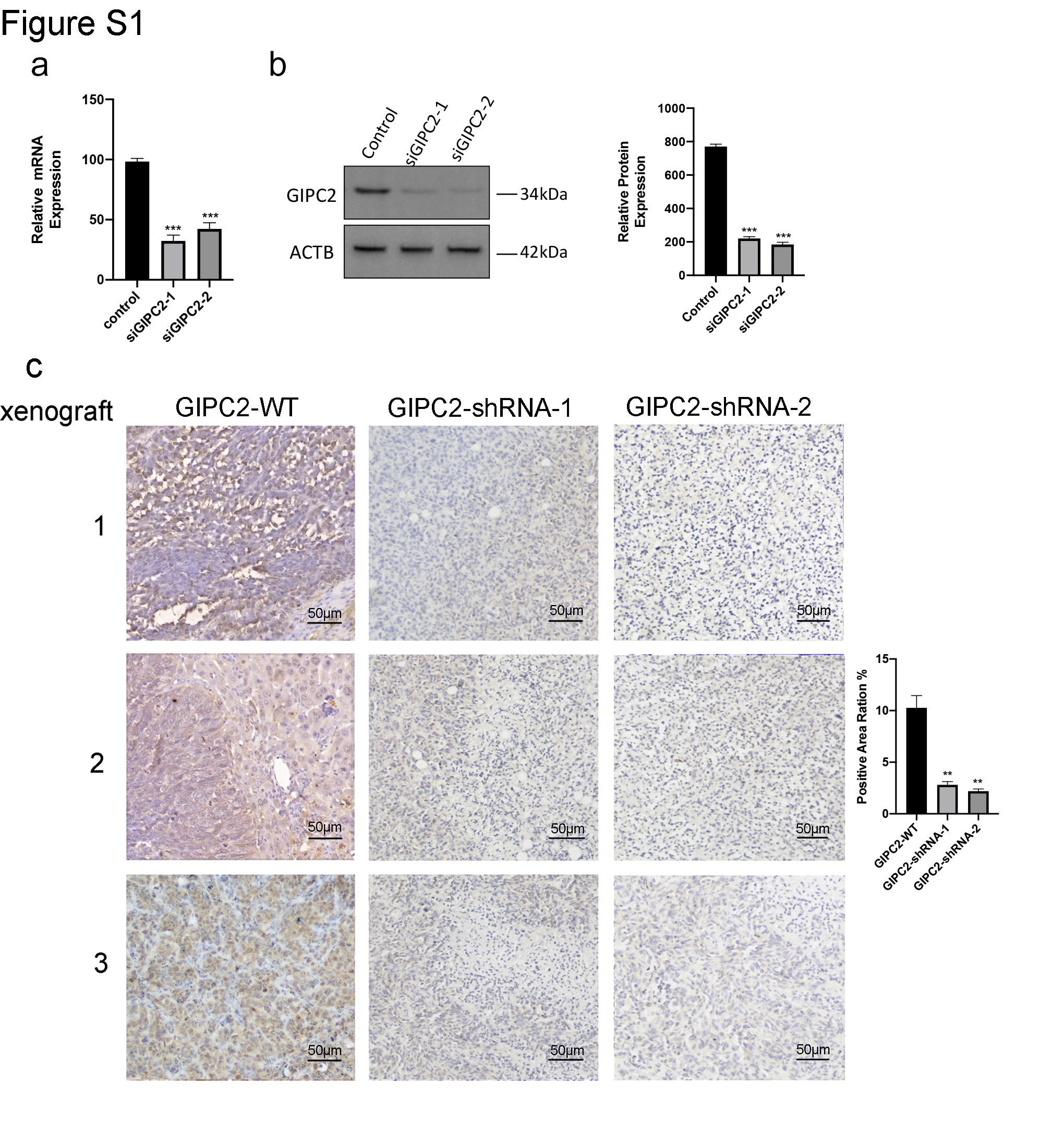

Supplement: Supplementary file 2 — Supplementary Figure S1 [file 41388_2022_2255_MOESM2_ESM.png]

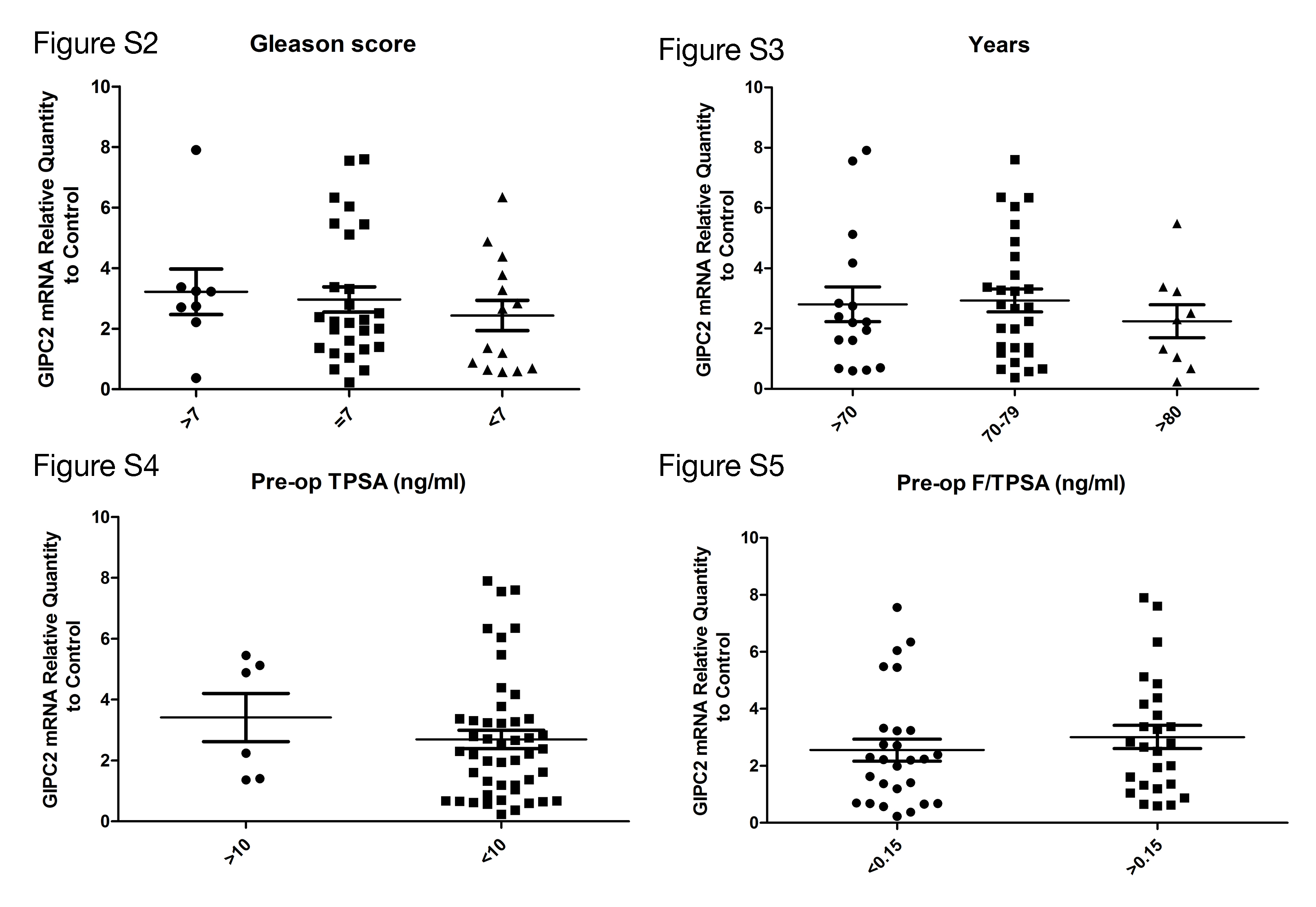

Supplement: Supplementary file 3 — Supplementary Figure S2–S5 [file 41388_2022_2255_MOESM3_ESM.png]

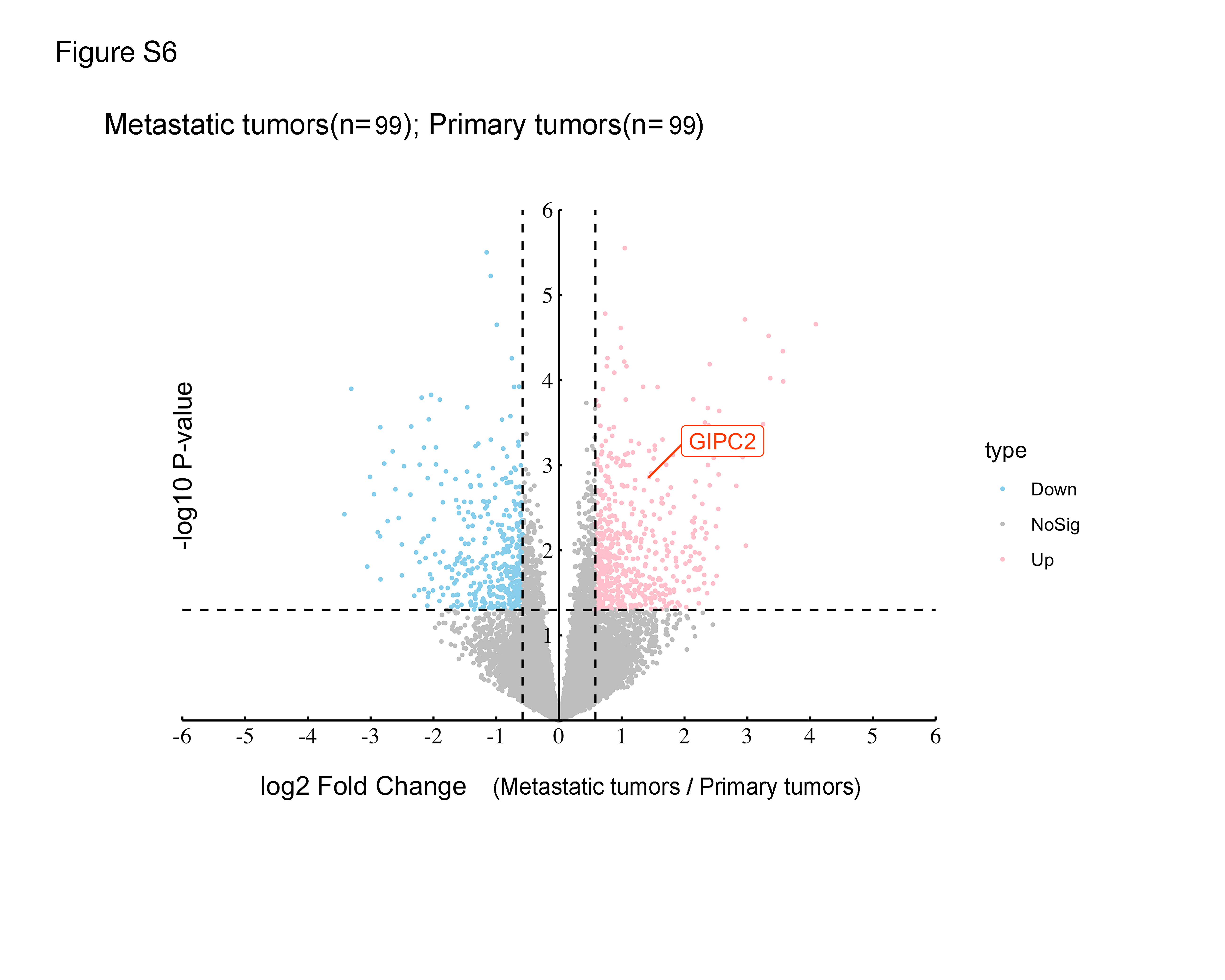

Supplement: Supplementary file 4 — Supplementary Figure S6 [file 41388_2022_2255_MOESM4_ESM.png]

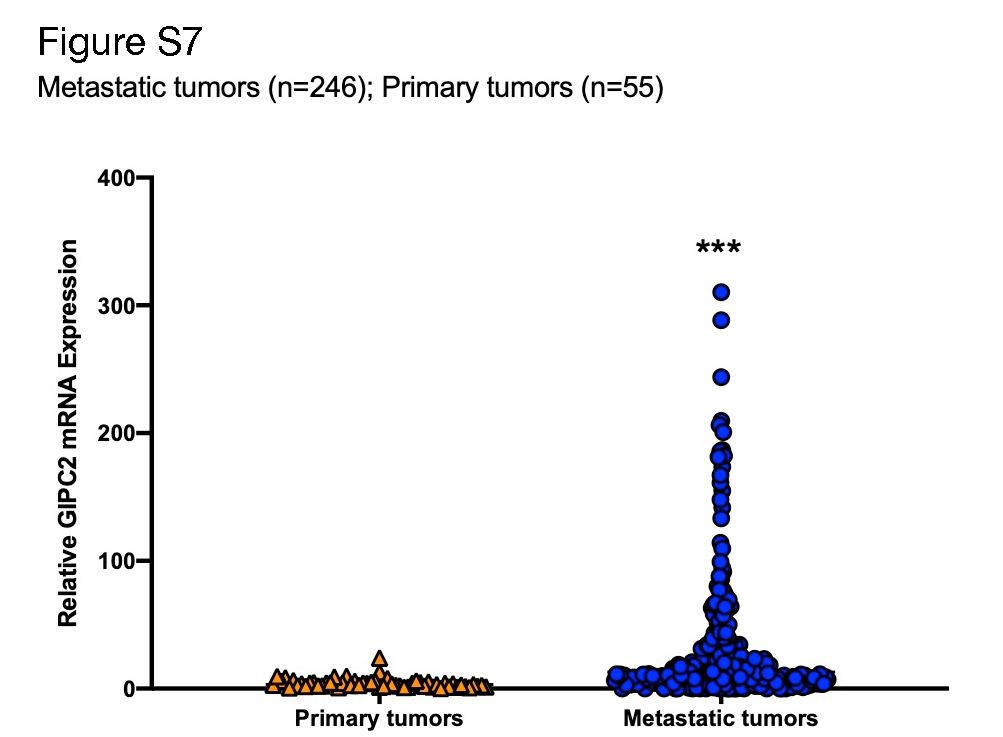

Supplement: Supplementary file 5 — Supplementary Figure S7 [file 41388_2022_2255_MOESM5_ESM.png]

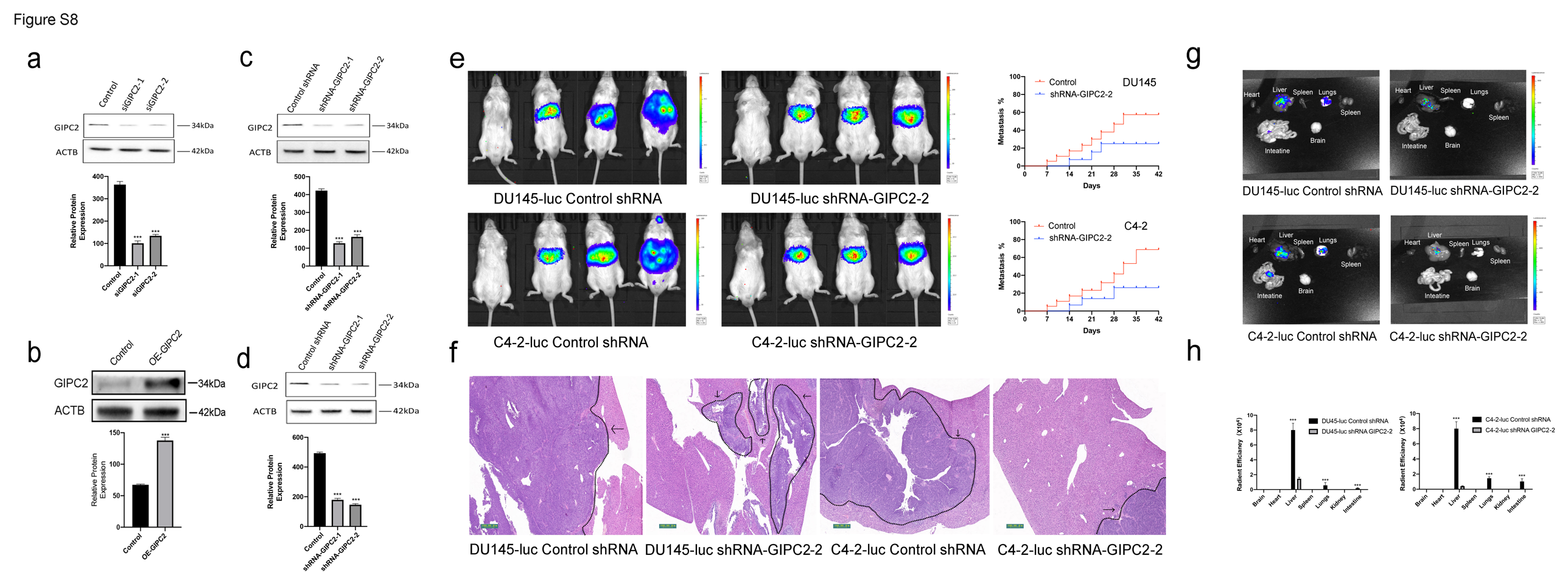

Supplement: Supplementary file 6 — Supplementary Figure S8 [file 41388_2022_2255_MOESM6_ESM.png]

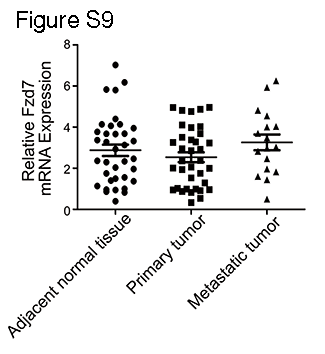

Supplement: Supplementary file 7 — Supplementary Figure S9 [file 41388_2022_2255_MOESM7_ESM.png]
