# Supplementary material for: GIPC2 interacts with Fzd7 to promote prostate cancer metastasis by activating WNT signaling
Source: Oncogene. 2022 Mar 28;41(18):2609–23. doi: 10.1038/s41388-022-02255-4 (PMC9054671; doi:10.1038/s41388-022-02255-4)
Supplement: Supplementary file 9 — Supplementary Table S2 [file 41388_2022_2255_MOESM9_ESM.docx]

**Table S2. Summary of clinical PCa samples**

| **Age** | **Number of patients** | **Evaluation of GIPC2 staining** | | |
| --- | --- | --- | --- | --- |
|  |  | **+1** | **+2** | **+3** |
| <70 | 16 |  |  |  |
| 70-79 | 26 |  |  |  |
| ≥80 | 7 |  |  |  |
|  |  |  |  |  |
| **Pre-op TPSA (ng/ml)** |  |  |  |  |
| <10 | 6 |  |  |  |
| ≥10 | 47 |  |  |  |
|  |  |  |  |  |
| **Pre-op F/TPSA (ng/ml)** |  |  |  |  |
| <0.15 | 27 |  |  |  |
| ≥0.15 | 26 |  |  |  |
|  |  |  |  |  |
| **Gleason score** |  |  |  |  |
| <7 | 8 | 8 |  |  |
| =7 | 27 | 5 | 19 | 3 |
| >7 | 14 |  | 7 | 7 |
|  |  |  |  |  |
| **Tumor status** |  |  |  |  |
| **Primary tumors** | 36 | 27 | 8 | 1 |
| **Metastatic tumors** | 17 | 0 | 4 | 13 |
