# Supplementary material for: GIPC2 interacts with Fzd7 to promote prostate cancer metastasis by activating WNT signaling
Source: Oncogene. 2022 Mar 28;41(18):2609–23. doi: 10.1038/s41388-022-02255-4 (PMC9054671; doi:10.1038/s41388-022-02255-4)
Supplement: Supplementary file 13 — Supplementary Table S6 [file 41388_2022_2255_MOESM13_ESM.docx]

Table S6 The average FRET efficiency (E) after photobleaching

|  | Positive control | Negative control | GIPC2-PDZ-Fzd7 | GIPC2-GH1-Fzd7 | GIPC2-GH2-Fzd7 |
| --- | --- | --- | --- | --- | --- |
| Mean E% | 22.01 | 2.21 | 25.11 | 2.25 | 2.60 |
| SD | 3.19 | 0.53 | 2.96 | 0.63 | 0.83 |
